# Identifying forces that stabilize the oligomeric state of bacterial homologs of neurotransmitter transporters

**DOI:** 10.1186/2050-6511-13-S1-A59

**Published:** 2012-09-17

**Authors:** Kumaresan Jayaraman, Azmat Sohail, Michael Freissmuth, Harald H Sitte, Thomas Stockner

**Affiliations:** 1 Institute of Pharmacology, Center for Physiology and Pharmacology, Medical University Vienna, 1090 Vienna, Austria

## Background

Neurotransmitter transporters of the SLC1 and SCL6 family are found on presynaptic neurons and on glia cells. The function of these transporters is the termination of neurotransmission by the rapid removal of the neurotransmitter molecules from the synaptic cleft. These transporters couple substrate transport to ion gradients of sodium and chloride. Almost all of the eucaryotic transporters have been described to function as oligomers. However, the forces stabilizing the oligomeric state are not well understood. No crystal structures of eukaryotic transporters are available, but recently crystal structures of bacterial homologs thereof have been solved: GltPh (SLC 1 family) was found as a trimer, LeuT (SLC6 family) was crystallized as a dimer. These homologous crystal structures allow rationalizing on the driving forces that stabilize the eukaryotic counterparts.

## Methods

The crystal structures of LeuT and GltPh were obtained from the Protein Data Bank (PDB). We identified the interfaces between the protomers and analyzed hydrogen bonding, hydrophobic and hydrophilic interactions as well as size and width of the interface area.

## Results

We investigated the protein-protein interfaces between the transporter protomers and identified the dominant forces that stabilize the oligomer. These consist of hydrophobic interactions between the aliphatic side chains within the interface and of polar interactions by hydrogen bonds between hydroxyl groups.

## Conclusions

The contributions of different forces to the stability of oligomer assemblies vary between proteins. While the hydrophobic mismatch is a prominent contributor to the stability of the GltPh transporter, it plays a minor role for LeuT, where helix packing and aromatic interactions seem to dominate.

